# Multiple sclerosis is not associated with an increased risk for severe COVID-19: a nationwide retrospective cross-sectional study from Germany

**DOI:** 10.1186/s42466-021-00143-y

**Published:** 2021-08-16

**Authors:** Daniel Richter, Simon Faissner, Dirk Bartig, Lars Tönges, Kerstin Hellwig, Ilya Ayzenberg, Christos Krogias, Ralf Gold

**Affiliations:** 1 Department of Neurology, St. Josef-Hospital, Ruhr-University Bochum, Bochum, Germany; 2 Medical Faculty, Ruhr-University Bochum, Bochum, Germany; 3 Center for Protein Diagnostics (ProDi), Ruhr University Bochum, Bochum, Germany; 4 Department of Neurology, I.M. Sechenov First Moscow State Medical University, Moscow, Russia

**Keywords:** Multiple sclerosis, COVID-19, SARS-CoV-2, Germany

## Abstract

**Background:**

Since the coronavirus disease 2019 (COVID-19) has risen, several risk factors have been identified, predicting a worse outcome. It has been speculated that patients with Multiple sclerosis (MS) have an increased risk for a severe course of COVID-19 due to a suspected higher vulnerability. Therefore, we aimed to analyze the impact of comorbid MS on the outcome of patients with COVID-19 in Germany.

**Methods:**

We conducted a retrospective cross-sectional study using the administrative database of all hospitalized patients diagnosed with PCR-confirmed COVID-19 (*n* = 157,524) in Germany during 2020. The cohort was stratified according to the presence (*n* = 551) or absence (*n* = 156,973) of comorbid MS, including discrimination of MS subtypes. Primary outcome measures were admission to the intensive care unit (ICU), use of invasive or non-invasive ventilation, and in-hospital mortality. Differences were investigated using rates and odds ratios as estimates. Pooled overall estimates, sex-stratified estimates, age-group stratified estimates, and MS subtype stratified estimates were calculated for all outcomes under the random-effects model.

**Results:**

Among 157,524 patients hospitalized with COVID-19, 551 had a concurrent MS diagnosis (0.3%). Overall, univariate analysis showed lower rates of ICU admission (17.1% versus 22.7%, *p* < 0.001), lower use of ventilation (9.8% versus 14.5%, *p* < 0.001) and lower in-hospital mortality (11.1% versus 19.3%, *p* < 0.001) among COVID-19 patients with comorbid MS. This finding was stable across the subgroup analysis of sex and MS subtype but was attenuated by age-stratification, confirming equal odds of in-hospital mortality between COVID-19 patients with and without MS (log OR: 0.09 [95% CI: − 0.40, 0.59]).

**Conclusions:**

Although there might be differences in risk within the MS patients’ population, this large-scale nationwide analysis found no evidence for a worse outcome of COVID-19 in patients with comorbid MS compared to non-MS individuals.

**Supplementary Information:**

The online version contains supplementary material available at 10.1186/s42466-021-00143-y.

## Background

The coronavirus disease 2019 (COVID-19) pandemic has spread rapidly, threatening global health [1]. The severe acute respiratory coronavirus 2 (SARS-CoV-2) high virulence can cause severe pneumonia with extensive lung inflammation that has caused innumerable victims worldwide. Since the beginning of the pandemic, there have been global efforts to gather information on the risk of infection and the specific consequences for patients with Multiple sclerosis (MS) [2, 3]. MS is the most common demyelinating disease of the central nervous system (CNS). The immunopathogenesis of MS involves CNS inflammation, including autoreactive lymphocytes, resulting in immunomodulating and immunosuppressive treatment approaches [4–6]. It has been speculated that patients with MS are at higher risk for a severe course of COVID-19, although robust evidence is lacking. Nevertheless, such assumptions have led to uncertainties in the MS community [7].

Different risk factors for worse outcomes of COVID-19 have been identified in the general population, including older age, male sex, and other comorbid conditions, such as hypertension, diabetes, and chronic obstructive pulmonary disease [8–11]. These comorbidities are also common in patients with Parkinson’s disease [12] or stroke, where comorbid COVID-19 has been associated with high in-hospital mortality [13]. Various reports and studies in patients with MS have evolved age and disability as important risk factors for a more severe course of COVID-19 within the MS patients’ population [3, 14–16]. However, there is lacking knowledge about the general role of comorbid MS as a factor influencing the outcome in COVID-19 patients compared to non-MS individuals.

Therefore, this study aimed to investigate the impact of comorbid MS on the outcome of patients with COVID-19 using comprehensive administrative diagnosis-related group (DRG) data from Germany.

## Methods

### Data source and study sample

The data that support the findings of this study are available from the corresponding author upon reasonable request. This is a German retrospective cross-sectional study using the administrative diagnosis-related group (DRG) database (Data transmission according to §21 KHEntgG and §24 para. 2 KHG; official data on file, source: Institut für das Entgeltsystem im Krankenhaus, InEK, www.g-drg.de). In Germany, all inpatient cases are encoded according to ICD-10-GM and operating and procedure keys (OPS) issued by the Federal Institute for Drugs and Medical Devices (BfArM). We included all patients hospitalized in Germany between January 1, 2020, to December 31, 2020, with the ICD-10 diagnosis of U07.1 (COVID-19, PCR-confirmed, *n* = 157,524). This cohort was divided into patients with (*n* = 551) and without (*n* = 156,973) comorbid MS defined by concurrent ICD-10 diagnosis of G35.1- (relapsing-remitting MS, *n* = 106), G35.2- (primary progressive MS, *n* = 76), G35.3- (secondary progressive MS, *n* = 99), and G35.9 (unspecified MS, *n* = 270). Patients being transferred once or multiple times from one hospital to another were censored appropriately to avoid multiple counting cases (excluding “discharge key 06”). The patient and outcome identification process of this study is given in the online supplement.

### Outcomes

Primary outcomes were admission to the intensive care unit (ICU), use of ventilation, and in-hospital mortality among COVID-19 patients with and without a concurrent main or secondary diagnosis of MS. The use of ventilation was defined by invasive or non-invasive ventilation for at least 1 h. In-hospital mortality was obtained using discharge key 07 (death during hospital stay).

### Statistical analysis

For descriptive analysis, results are reported as mean and standard deviation (SD) for continuous variables and absolute numbers and percentages for categorical variables. Univariate analysis was performed with the chi-squared test (χ2) and t-test, respectively. Subgroup analysis of the primary outcomes was investigated using odds ratio (OR) as estimate to compare the likelihoods between COVID-19 patients with and without a concurrent MS diagnosis. Pooled overall estimates, sex-stratified estimates, age-group stratified estimates, and MS subtype stratified estimates were calculated for all outcomes under the random-effects model (DerSimonian-Laird). Differences of subgroup-stratified estimates within and in-between the primary outcome analysis were investigated with the Cochran test for heterogeneity and I^2^ statistics. *P* < 0.05 was set as the level of statistical significance. Calculations were performed with the Stata Statistical Software Release 17 for Mac (StataCorp LP, College Station, TX).

## Results

### Demographics

Among 157,524 patients hospitalized with COVID-19, 551 had a concurrent diagnosis of MS (0.3%). There was a higher percentage of women in the cohort of patients with comorbid MS (62.8% versus 48.2%, *p* < 0.001). Age was lower in patients with comorbid MS (60.6 ± 4.5 versus 66.9 ± 7.3, p < 0.001), in line with a lower rate of patients over 60 years (52.5% versus 68.2%, *p* < 0.001). COVID-19 patients with comorbid RRMS patients were younger compared to those with comorbid PPMS (51.2 ± 3.4 versus 61.6 ± 4.7, *p* < 0.001), SPMS (63.5 ± 6.2, *p* < 0.001), or unspecified MS (62.2 ± 4.7, *p* < 0.001).

### Univariate analysis

Univariate analysis of the primary outcome measures revealed lower rates for ICU admission (17.1% versus 22.7%, *p* < 0.001), lower use of ventilation (9.8% versus 14.5%, *p* < 0.001), and lower in-hospital mortality (11.1% versus 19.3%, *p* < 0.001) among COVID-19 patients with comorbid MS (Table 1).

**Table 1:** General characteristics of COVID-19 patients stratified by the presence of comorbid MS

	Comorbid MS (***n*** = 551)	No comorbid MS (***n*** = 156,973)	***P***-Value
**Female (n, %)**	346, 62.8	75,593, 48.2	< 0.001
**Male (n, %)**	205, 37.2	81,380, 51.8	< 0.001
**Age (mean ± SD)**	60.3 ± 4.5	66.9 ± 7.3	< 0.001
**Age ≥ 60 years (n, %)**	289, 52.5	106,986, 68.2	< 0.001
**Admission to ICU (n, %)**	94, 17.1	35,564, 22.7	< 0.001
**Ventilation (n, %)**	54, 9.8	22,692, 14.5	< 0.001
**In-hospital mortality (n, %)**	61, 11.1	30,246, 19.3	< 0.001

### Subgroup analysis

**Fig. 1:**
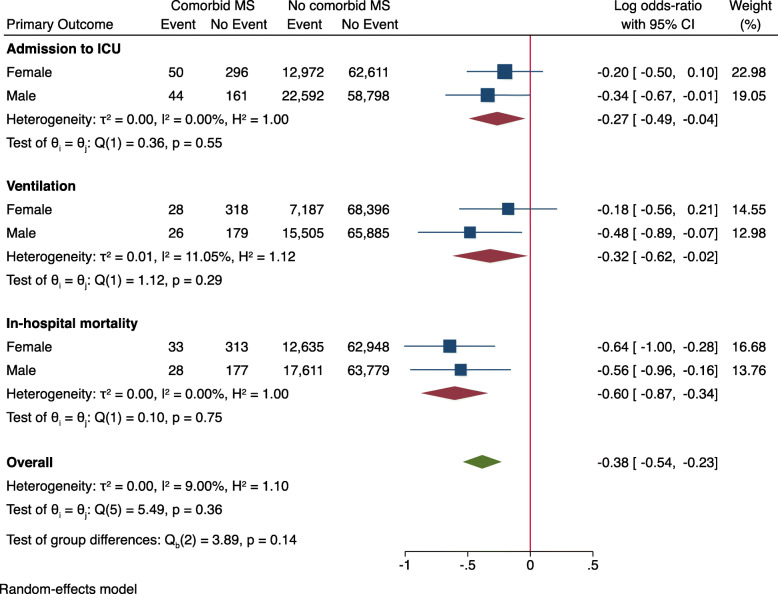
Primary outcome parameters stratified by sex. Log odds-ratio < 0 indicate lower risk for patients with comorbid MS. Abbreviations: ICU = intensive care unit

**Fig. 2:**
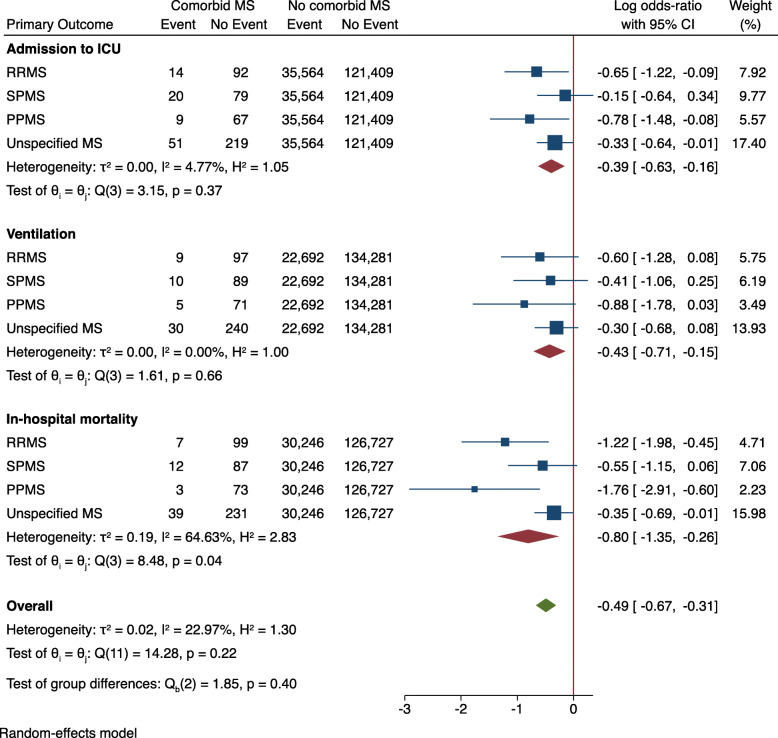
Primary outcome parameters stratified by MS-subtype. Log odds-ratio < 0 indicates a lower risk for patients with comorbid MS. Abbreviations: ICU = intensive care unit

**Fig. 3:**
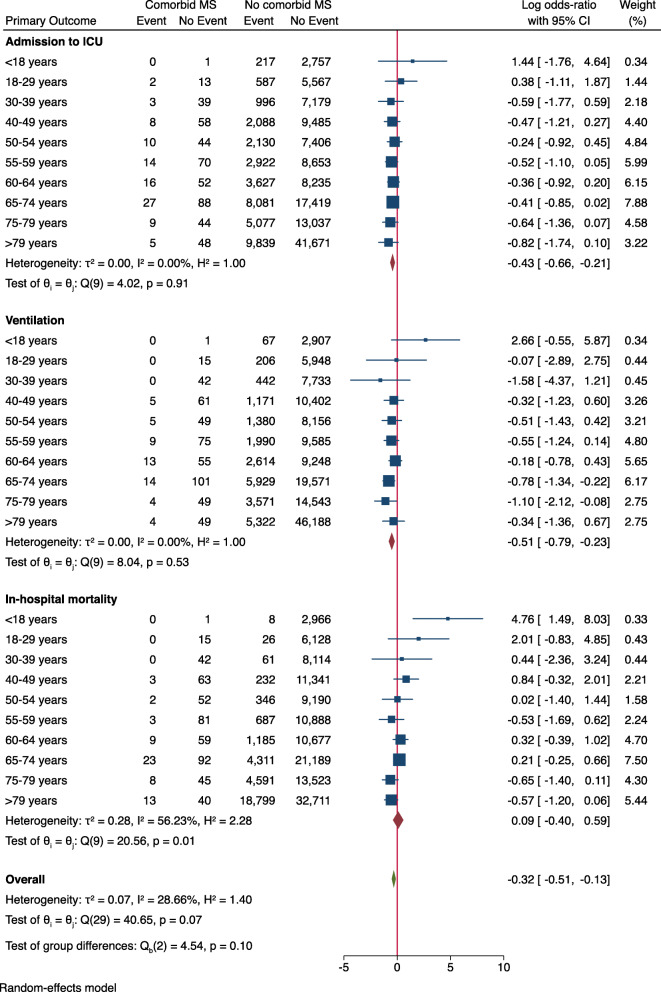
Primary outcome parameters stratified by age-group. Log odds-ratio < 0 indicates a lower risk for patients with comorbid MS. Abbreviations: ICU = intensive care unit

Sex- and MS subtype-stratified analysis demonstrated lower OR in patients with comorbid MS for all primary outcome measures (Figs. 1 and 2). When stratified by age groups, there was no significant difference in the OR for in-hospital mortality between patients with and without comorbid MS (log OR: 0.09 [95% CI: − 0.40, 0.59], Fig. 3). Age-stratified analysis was in line with the sex and MS subtype stratified findings, showing lower OR of admission to ICU (log OR: -0.43 [95% CI: − 0.66, − 0.21]) and use of invasive or non-invasive ventilation (log OR: -0.51 [95% CI: − 0.79, − 0.23]) in patients with comorbid MS.

## Discussion

This is the first large-scale nationwide analysis from Germany investigating the outcome of COVID-19 in patients with comorbid MS. Overall, 0.3% of all hospitalized COVID-19 patients had a concurrent MS diagnosis. We found no evidence for a worse outcome of COVID-19 in patients with MS compared to non-MS patients.

**Fig. 4:**
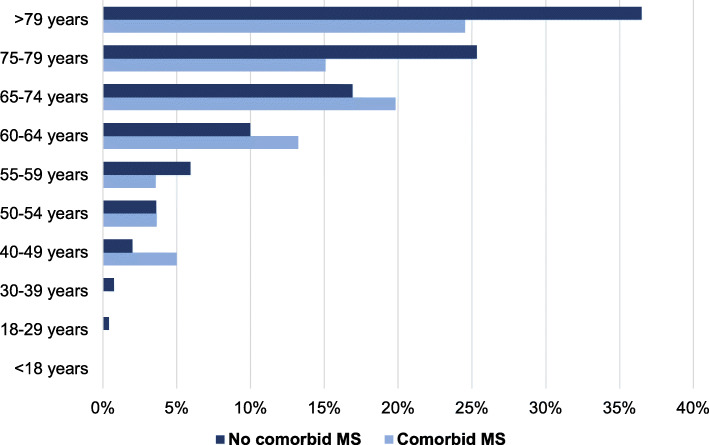
In-hospital mortality rates of COVID-19 patients stratified by age and the presence of comorbid MS. *P*-Value > 0.05 for all age-group comparisons among patients with and without comorbid MS

Indeed, all primary outcome rates were lower in MS patients compared to the non-MS cohort. The overall in-hospital mortality rate of COVID-19 patients with comorbid MS was 10.9%. Although the lower odds for MS patients were stable over the subgroup analysis of MS subtypes and sex, the age-group analysis revealed no difference between patients with and without comorbid MS in terms of in-hospital mortality. This is the most reasonable explanation for the observed discrepancies of the pooled univariate analysis, as the MS patients in our study were younger than the non-MS individuals. In general, higher age is known to be an essential risk factor for a severe course of COVID-19 that was strongly associated with higher mortality. Recent data from England, Geneva in Switzerland, and Spain confirmed age as an independent predictor of dying from a SARS-CoV-2 infection [17–20]. The colleagues found an increase in the infection fatality ratio with higher age [17]. Although there is limited in-between comparability of these data due to different age-group definitions, the data from Geneva indicates an infection fatality ratio of 5.6% in patients older than 64 years, England report 11.6% in patients older than 74 years and Spain reports 7.2% in patients older than 79 years. All countries observed an infection fatality ratio far below 1% in SARS-CoV-2 antibody-positive individuals under 50 years. The infection fatality ratios of these different studies were calculated based on the serological status (presence of SARS-CoV-2 antibodies). Therefore, they are not directly transferable to our findings because our data refer to all hospitalized patients with laboratory-confirmed COVID-19 in Germany. However, we observed an equal dynamic of the in-hospital mortality that mirrors the observations of the colleagues. We also found that the in-hospital mortality of COVID-19 patients increased with age, which we document for both groups, patients with and without comorbid MS (Fig. 4).

Besides age, the male sex was also associated with higher mortality among individuals with SARS-CoV-2 infection [17]. This also applies to patients with comorbid MS, as we also found a higher likelihood to die in male compared to female COVID-19 patients with comorbid MS (Online supplement). Although there was a higher frequency of women in our cohort of COVID-19 patients with comorbid MS, the sex-stratified analysis confirmed lower odds for all outcome measures, indicating age rather than sex as the major confounding factor for the observed differences between COVID-19 patients with and without comorbid MS.

The OR for admission to ICU and use of invasive or non-invasive ventilation were lower in patients with comorbid MS across all subgroup stratifications. Although the effect was attenuated in the age-group-stratified analysis, it remained statistically significant. The reason for this is speculative as there is a lack of information regarding other clinical and comorbid conditions, which is the major limitation of this study. Considering the high heterogenicity in the in-hospital mortality analysis across MS subtypes, such information is particularly important. We had no information about the use of immunosuppressive medication and the degree of disability of the study cohort. Unfortunately, this data is not consistently coded in the DRG database, making it impossible to account for it. Furthermore, admission to ICU might differ between hospitals that could also have biased the findings.

Besides age, Black race, cardiovascular comorbidities, and recent treatment with corticosteroids, an increased disability has recently been linked with clinical severity of COVID-19 in patients with comorbid MS [Salter 2021]. The German nursing institutions were almost sealed off and had to comply with strict quarantine rules during 2020. Additionally, highly disabled MS patients may have been more prudent and therefore better protected against a SARS-CoV-2 infection in- and outside of these institutions, resulting in the overall lower OR of the predefined outcomes. This hypothesis is also supported by the substantial decline in hospitalizations of MS patients with a progressive disease course in Germany, that was most remarkable during the first wave the pandemic in March 2020 [21].

Another punt for speculation is that PPMS patients had a lower OR for in-hospital mortality compared to SPMS and RRMS patients, although the age of these subgroups was similar or younger. As the information is not registered, this dataset cannot answer whether this is due to a lower use of immunosuppressive drugs in patients with PPMS. Despite the lack of confounding factors, this large-scale nationwide analysis used comprehensive administrative data from Germany based on the documented diagnoses and procedures in the G-DRG system. The administrative data have high quality and accuracy because registration of all inpatient cases and procedures is a prerequisite to getting financial compensation. Although generally not all coding of, e.g., secondary diagnoses, lead to higher reimbursement, all codes within the G35.- category are relevant for determining the case severity. In this respect, systematic under-determination cannot be ruled out, but it is unlikely.

Nevertheless, reimbursement by coding within the G35.- category is irrespective of the MS subtype coding. This might be reflected by the fact that nearly half of the MS patients were coded with an unspecified disease course. Therefore, another explanation for the low mortality in the PPMS group might be a wrong attribution. However, the coding is closely controlled by medical services of the medical health insurances to ensure the proper financial compensation for all German hospitals. The almost complete coverage of all hospitalized patients in Germany with a shallow risk of missing patients or double coding procedures is an essential strength of this study.

## Conclusion

This study shows that simply having MS does not increase the likelihood of a severe course of COVID-19 compared to patients without comorbid MS. Nevertheless, there might be differences in risk within the MS patients’ population. Therefore, the knowledge of MS-associated risk factors is vital to help clinicians identify patients at higher risk for a severe course of COVID-19. Future studies should also question the incidence and impact of Long-COVID syndromes in patients with comorbid MS.

## Supplementary Information

**Additional file 1: Figure S1.** Patient and outcome identification process.

**Additional file 2: Figure S2.** In-hospital mortality of COVID-19 patients with comorbid MS stratified by age and sex. Log odds-ratio > 0 indicates a higher risk for males.

## Data Availability

Data according to §21 KHEntgG and §24 para. 2 KHG; official data on file, source: Destatis, www.destatis.de.

## Abbreviations

MS: Multiple sclerosis
COVID-19: Coronavirus disease 2019
SARS-CoV-2: Severe acute respiratory coronavirus 2
G-DRG: German Diagnosis-Related Groups
ICU: Intensive care unit
