# Supplementary material for: Multiple sclerosis is not associated with an increased risk for severe COVID-19: a nationwide retrospective cross-sectional study from Germany
Source: Neurol Res Pract. 2021 Aug 16;3:42. doi: 10.1186/s42466-021-00143-y (PMC8364944; doi:10.1186/s42466-021-00143-y)
Supplement: Supplementary file 1 — Additional file 1: Figure S1. Patient and outcome identification process. [file 42466_2021_143_MOESM1_ESM.pdf]

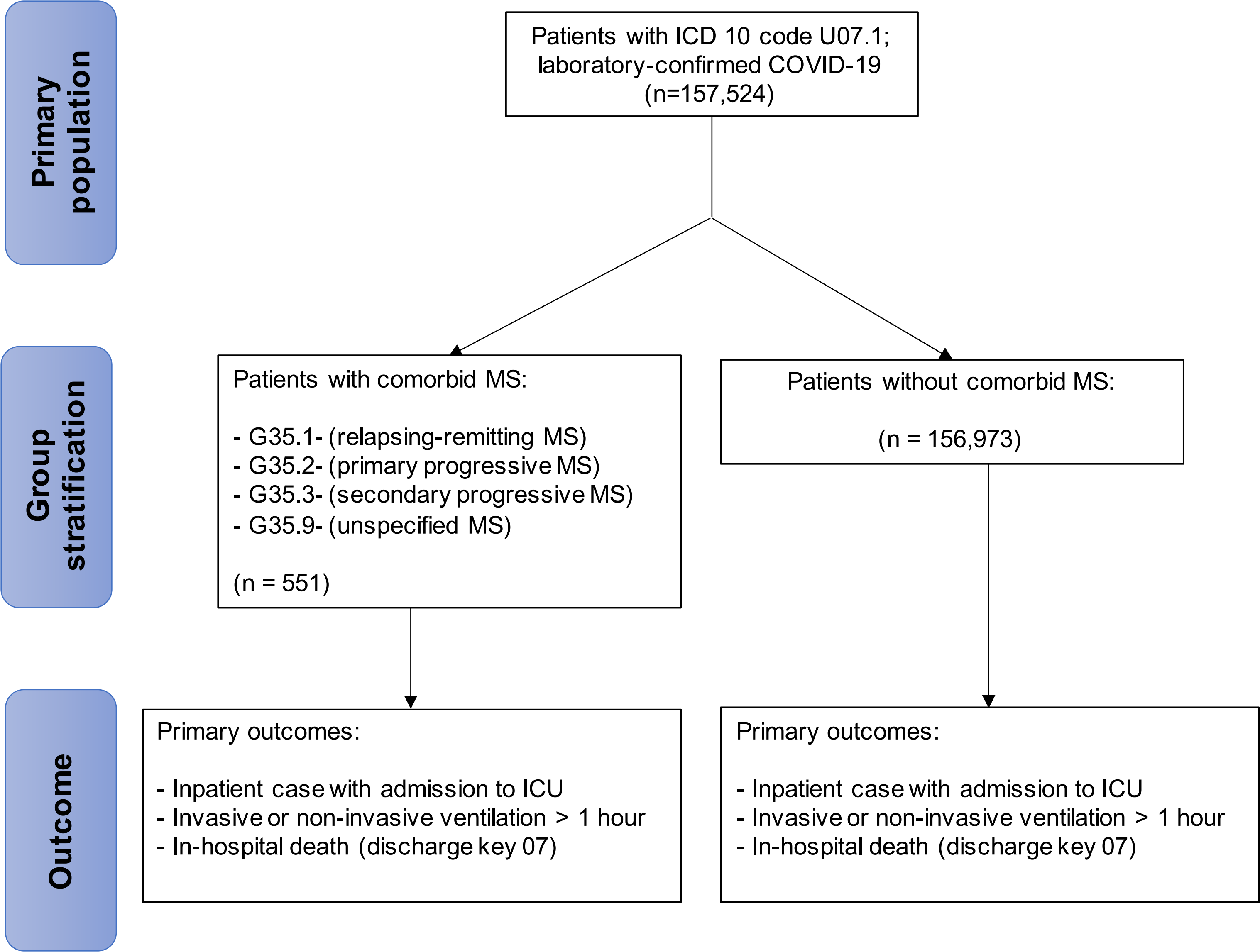
Primary population
Patients with ICD 10 code U07.1; laboratory-confirmed COVID-19 (n=157,524)
Group stratification
Patients with comorbid MS:
- G35.1- (relapsing-remitting MS)
- G35.2- (primary progressive MS)
- G35.3- (secondary progressive MS)
- G35.9- (unspecified MS)
(n = 551)
Patients without comorbid MS:
(n = 156,973)
Outcome
Primary outcomes:
- Inpatient case with admission to ICU
- Invasive or non-invasive ventilation > 1 hour
- In-hospital death (discharge key 07)
Primary outcomes:
- Inpatient case with admission to ICU
- Invasive or non-invasive ventilation > 1 hour
- In-hospital death (discharge key 07)
