# Supplementary figures and images for: Multiple sclerosis is not associated with an increased risk for severe COVID-19: a nationwide retrospective cross-sectional study from Germany
Source: Neurol Res Pract. 2021 Aug 16;3:42. doi: 10.1186/s42466-021-00143-y (PMC8364944; doi:10.1186/s42466-021-00143-y)

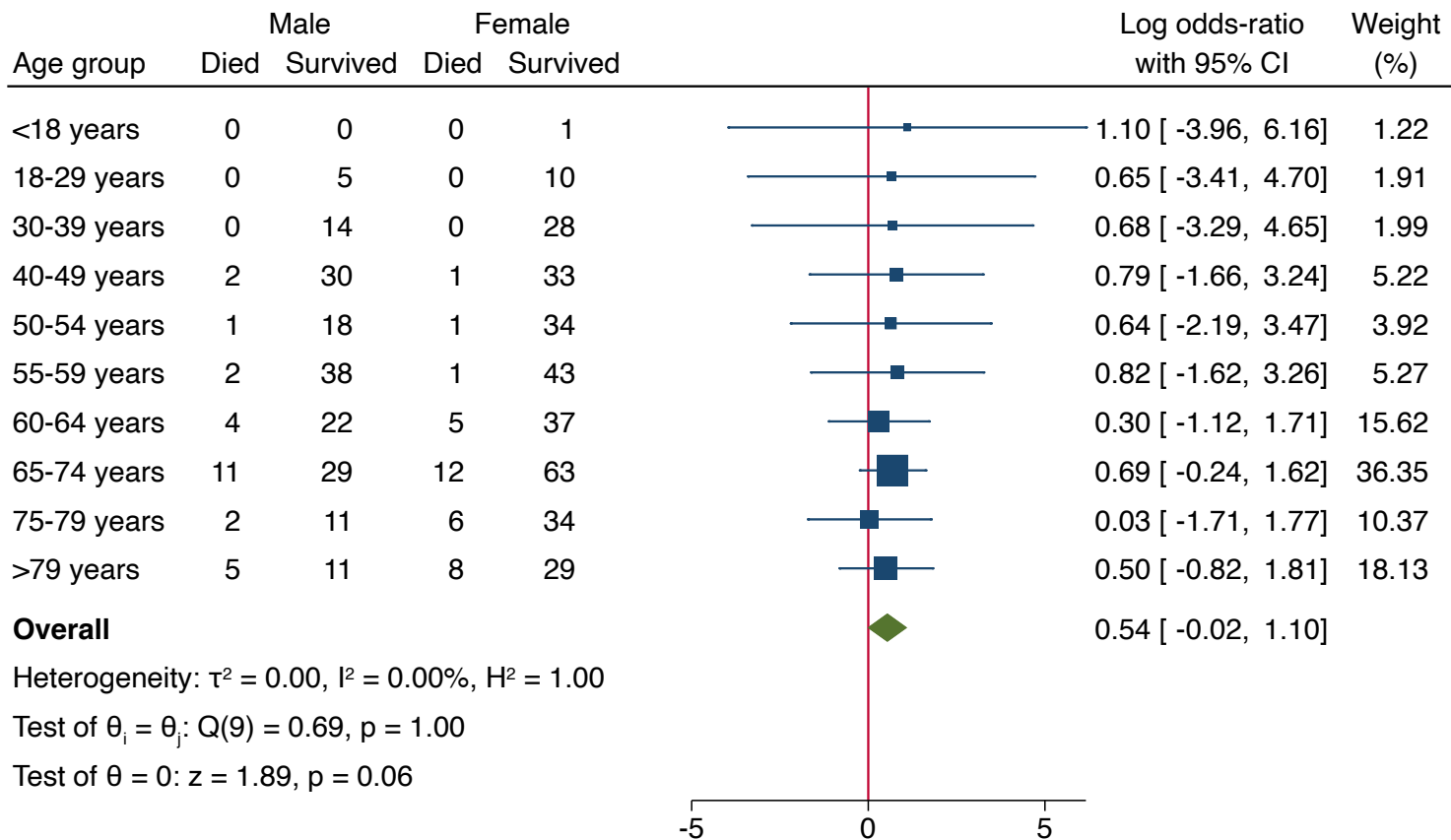

Random-effects model

Supplement: Supplementary file 2 — Additional file 2: Figure S2. In-hospital mortality of COVID-19 patients with comorbid MS stratified by age and sex. Log odds-ratio > 0 indicates a higher risk for males. [file 42466_2021_143_MOESM2_ESM.pdf]
